# Ablação de Fibrilação Atrial: Ainda Estamos Procurando a Melhor Opção?

**DOI:** 10.36660/abc.20250396

**Published:** 2025-08-06

**Authors:** Lucas Simonetto Faganello, Mauricio Pimentel

**Affiliations:** 1 Hospital de Clinicas de Porto Alegre Porto Alegre RS Brasil Hospital de Clinicas de Porto Alegre, Porto Alegre, RS – Brasil

**Keywords:** Fibrilação Atrial, Ablação

A fibrilação atrial (FA) está independentemente associada a riscos aumentados de mortalidade, acidente vascular cerebral, insuficiência cardíaca, comprometimento da qualidade de vida e declínio cognitivo. O controle do ritmo cardíaco demonstrou ser superior ao controle da frequência cardíaca na redução de desfechos cardiovasculares, com a ablação por cateter demonstrando maior eficácia do que a terapia medicamentosa antiarrítmica.^1 – 3^ Avanços contínuos em técnicas e tecnologias têm progressivamente estabelecido a ablação como um pilar fundamental no tratamento da FA.

Nos últimos anos, o mapeamento 3D e a ablação ponto a ponto evoluíram significativamente com a introdução de cateteres com sensor de força de contato e o desenvolvimento de métricas de qualidade da lesão, como o índice de ablação e o índice de tamanho da lesão. Essas melhorias se traduziram em melhores resultados, tempos de procedimento reduzidos e maior segurança.^3 – 5^ O isolamento das veias pulmonares continua sendo a pedra angular do tratamento invasivo da FA, sendo a ablação ponto a ponto por radiofrequência (RF) a técnica mais utilizada, apesar do surgimento de fontes de energia alternativas. O sucesso do procedimento depende da aplicação de lesões contínuas, irreversíveis e transmurais que circundem as veias pulmonares, preservando as estruturas adjacentes, como o esôfago e o nervo frênico.

A técnica de alta potência e curta duração (HPSD) surgiu como uma alternativa à ablação por RF convencional, com o objetivo de produzir lesões mais superficiais, porém mais amplas, aumentando o aquecimento resistivo e minimizando o aquecimento condutivo, reduzindo assim o tempo de ablação.^6^ Com base nesse conceito, a técnica de altíssima potência e curta duração (VHPSD) foi recentemente introduzida, fornecendo 90 watts em 4 segundos. Essa abordagem demonstrou um perfil de segurança favorável e altas taxas de isolamento na primeira passagem.^7^

A ablação por campo pulsado (ACP) é uma modalidade não térmica baseada em eletroporação irreversível, que utiliza pulsos elétricos ultracurtos e de alta energia para criar lesões transmurais permanentes, preservando os tecidos circundantes.^8^ A ausência de lesão esofágica e suas complicações potencialmente catastróficas têm gerado considerável interesse na comunidade eletrofisiológica. Dados de registros sugerem que a ACP proporciona desfechos favoráveis com baixas taxas de complicações.^9 , 10^ Em um ensaio clínico randomizado comparando diretamente a ACP com a ablação por RF convencional, não foram encontradas diferenças significativas entre as duas técnicas.^11^ Além disso, um estudo observacional recente comparando ACP e HPSD mostrou tempos de procedimento mais curtos e menores taxas de recorrência de FA com ACP.^12^

Nesta edição dos Arquivos Brasileiros de Cardiologia, uma metanálise elegante compara os resultados de ACP e VHPSD para ablação de FA.^13^ A análise inclui quatro estudos observacionais abrangendo um total de 605 pacientes. Ambas as técnicas demonstraram taxas de sucesso comparáveis e taxas de complicações semelhantes. A ACP foi associada a um tempo total de procedimento mais curto, mas a uma duração de fluoroscopia mais longa. Este estudo é clinicamente relevante, pois fornece uma comparação oportuna entre duas estratégias contemporâneas de ablação. No entanto, uma limitação importante é que apenas estudos observacionais foram incluídos; nenhum ensaio clínico randomizado comparou diretamente ACP e VHPSD. Esses achados estão em linha com os de outra metanálise recente que aborda a mesma questão.^14^

A ablação por cateter está agora firmemente estabelecida como a estratégia de controle de ritmo mais eficaz para FA. O desenvolvimento contínuo de novas técnicas e a busca pela estratégia de ablação ideal permanecem altamente relevantes. Há uma clara necessidade de ensaios clínicos randomizados robustos comparando essas tecnologias. Além disso, considerando as variações de custo e acessibilidade, análises de custo-efetividade serão essenciais para orientar a tomada de decisões. Nesse contexto, em que a Sociedade Brasileira de Arritmias Cardíacas obteve recentemente aprovação para ablações complexas no Sistema Único de Saúde (SUS), definir a estratégia de ablação mais eficaz será fundamental para a formulação de políticas de saúde pública e a otimização da alocação de recursos.^15^

## References

[1] Kirchhof P, Camm AJ, Goette A, Brandes A, Eckardt L, Elvan A (2020). Early Rhythm-Control Therapy in Patients with Atrial Fibrillation. N Engl J Med.

[2] Osorio J, Miranda-Arboleda AF, Velasco A, Varley AL, Rajendra A, Morales GX (2024). Real-World Data of Radiofrequency Catheter Ablation in Paroxysmal Atrial Fibrillation: Short- and Long-Term Clinical Outcomes from the Prospective Multicenter REAL-AF Registry. Heart Rhythm.

[3] Ternes CMP, Rohde LE, Forno AD, Lewandowski A, Nascimento HG, Odozynski G (2025). The Southern Brazilian Registry of Atrial Fibrillation (SBR-AF Registry): Predictors of Atrial Arrhythmia Recurrence after First-Time Catheter Ablation. Arq Bras Cardiol.

[4] Afzal MR, Chatta J, Samanta A, Waheed S, Mahmoudi M, Vukas R (2015). Use of Contact Force Sensing Technology during Radiofrequency Ablation Reduces Recurrence of Atrial Fibrillation: A Systematic Review and Meta-Analysis. Heart Rhythm.

[5] Hussein A, Das M, Riva S, Morgan M, Ronayne C, Sahni A (2018). Use of Ablation Index-Guided Ablation Results in High Rates of Durable Pulmonary Vein Isolation and Freedom from Arrhythmia in Persistent Atrial Fibrillation Patients: The PRAISE Study Results. Circ Arrhythm Electrophysiol.

[6] Leshem E, Zilberman I, Tschabrunn CM, Barkagan M, Contreras-Valdes FM, Govari A (2018). High-Power and Short-Duration Ablation for Pulmonary Vein Isolation: Biophysical Characterization. JACC Clin Electrophysiol.

[7] Heeger CH, Sano M, Popescu SȘ, Subin B, Feher M, Phan HL (2023). Very High-Power Short-Duration Ablation for Pulmonary Vein Isolation utilizing a Very-Close Protocol-the FAST AND FURIOUS PVI Study. Europace.

[8] Reddy VY, Neuzil P, Koruth JS, Petru J, Funosako M, Cochet H (2019). Pulsed Field Ablation for Pulmonary Vein Isolation in Atrial Fibrillation. J Am Coll Cardiol.

[9] Chun KJ, Miklavčič D, Vlachos K, Bordignon S, Scherr D, Jais P (2024). State-of-the-Art Pulsed Field Ablation for Cardiac Arrhythmias: Ongoing Evolution and Future Perspective. Europace.

[10] Scanavacca M, Pisani C (2024). Impact of Pulsed Field Ablation on Atrial Fibrillation. Arq Bras Cardiol.

[11] Reddy VY, Gerstenfeld EP, Natale A, Whang W, Cuoco FA, Patel C (2023). Pulsed Field or Conventional Thermal Ablation for Paroxysmal Atrial Fibrillation. N Engl J Med.

[12] Santos RR, Amador R, Santos PG, Matos D, Rodrigues G, Carmo J (2025). Atrial Fibrillation Catheter Ablation: Electroporation Against High-Power Short Duration Radiofrequency. Arq Bras Cardiol.

[13] Iqbal A, Amador WFO, Cevallos-Cueva M, Ahmad M, Alarcón JAP, Diniz RV (2025). Pulsed Field Ablation Versus Very High-Power Short-Duration Radiofrequency Ablation in Atrial Fibrillation: A Systematic Review and Meta-Analysis. Arq Bras Cardiol.

[14] Bulhões E, Mazetto RASV, Vanio AL, Defante MLR, Feitoza L, Guida C (2025). Comparing Pulsed Field Ablation with Very High-Power and High-Power Short-Duration Radiofrequency Ablation for Atrial Fibrillation: A Systematic Review and Meta-Analysis. J Interv Card Electrophysiol.

[15] Brasil. Ministério da Saúde (2025). Diário Oficial da União.

