# Supplementary material for: Ablação de Fibrilação Atrial: Ainda Estamos Procurando a Melhor Opção?
Source: Arq Bras Cardiol. 2025 Aug 6;122(7):e20250396. [Article in Portuguese] doi: 10.36660/abc.20250396 (PMC12695039; doi:10.36660/abc.20250396)
Supplement: Translation

# Translation: Atrial Fibrillation Ablation: Are We Still Looking for the Best Shot?

**DOI:** 10.36660/abc.20250396i

**Authors:** Lucas Simonetto Faganello, Mauricio Pimentel

**Affiliations:** 1 Porto Alegre RS Brazil Hospital de Clinicas de Porto Alegre, Porto Alegre, RS – Brazil

**Keywords:** Atrial Fibrillation, Ablation

Atrial fibrillation (AF) is independently associated with increased risks of mortality, stroke, heart failure, impaired quality of life, and cognitive decline. Rhythm control has proven superior to rate control in reducing cardiovascular outcomes, with catheter ablation demonstrating greater efficacy than antiarrhythmic drug therapy.^1 – 3^Continuous advances in techniques and technologies have progressively established ablation as a cornerstone in the management of AF.

In recent years, 3D mapping and point-by-point ablation have evolved significantly with the introduction of contact force-sensing catheters and the development of lesion quality metrics, such as the Ablation Index and Lesion Size Index. These improvements have translated into better outcomes, reduced procedure times, and enhanced safety.^3 – 5^Pulmonary vein isolation remains the cornerstone of invasive AF treatment, with radiofrequency (RF) point-by-point ablation still being the most widely used technique despite the emergence of alternative energy sources. Procedural success relies on delivering continuous, irreversible, and transmural lesions encircling the pulmonary veins while sparing adjacent structures, such as the esophagus and phrenic nerve.

The high-power short-duration (HPSD) technique has emerged as an alternative to conventional RF ablation, aiming to produce more superficial but wider lesions by enhancing resistive heating while minimizing conductive heating, thereby shortening ablation time.^6^Building upon this concept, the very high-power short-duration (VHPSD) technique was recently introduced, delivering 90 watts over 4 seconds. This approach has demonstrated a favorable safety profile and high first-pass isolation rates.^7^

Pulsed-field ablation (PFA) is a non-thermal modality based on irreversible electroporation, which uses high-energy, ultra-short electrical pulses to create permanent transmural lesions while preserving surrounding tissues.^8^The absence of esophageal injury and its potentially catastrophic complications has generated considerable interest in the electrophysiology community. Registry data suggest that PFA provides favorable outcomes with low complication rates.^9 – 10^In a randomized clinical trial directly comparing PFA with conventional RF ablation, no significant differences were found between the two techniques.^11^Furthermore, a recent observational study comparing PFA and HPSD showed shorter procedure times and lower AF recurrence rates with PFA.^12^

In this issue of *Arquivos Brasileiros de Cardiologia* , an elegant meta-analysis compares the outcomes of PFA and VHPSD for AF ablation.^13^The analysis includes four observational studies comprising a total of 605 patients. Both techniques demonstrated comparable success rates and similar complication rates. PFA was associated with a shorter total procedure time but a longer fluoroscopy duration. This study is clinically relevant as it provides a timely comparison between two contemporary ablation strategies. However, a key limitation is that only observational studies were included; no randomized clinical trials have yet directly compared PFA and VHPSD. These findings are in line with those of another recent meta-analysis addressing the same question.^14^

Catheter ablation is now firmly established as the most effective rhythm control strategy for AF. The continuous development of novel techniques and the search for the optimal ablation strategy remain highly relevant. There is a clear need for robust randomized clinical trials comparing these technologies. Additionally, considering variations in cost and accessibility, cost-effectiveness analyses will be essential to guide decision-making. In this context, where the *Sociedade Brasileira de Arritmias Cardíacas* has recently succeeded in securing approval for complex ablations within the Brazilian public health system (SUS), defining the most effective ablation strategy will be pivotal for shaping public health policy and optimizing resource allocation.^15^
